# Stress-specific response of the p53-Mdm2 feedback loop

**DOI:** 10.1186/1752-0509-4-94

**Published:** 2010-07-12

**Authors:** Alexander Hunziker, Mogens H Jensen, Sandeep Krishna

**Affiliations:** 1 Center for Models of Life, Niels Bohr Institute, Copenhagen, Denmark; 2 National Centre for Biological Sciences, Bangalore, India

## Abstract

**Background:**

The p53 signalling pathway has hundreds of inputs and outputs. It can trigger cellular senescence, cell-cycle arrest and apoptosis in response to diverse stress conditions, including DNA damage, hypoxia and nutrient deprivation. Signals from all these inputs are channeled through a single node, the transcription factor p53. Yet, the pathway is flexible enough to produce different downstream gene expression patterns in response to different stresses.

**Results:**

We construct a mathematical model of the negative feedback loop involving p53 and its inhibitor, Mdm2, at the core of this pathway, and use it to examine the effect of different stresses that trigger p53. In response to DNA damage, hypoxia, etc., the model exhibits a wide variety of specific output behaviour - steady states with low or high levels of p53 and Mdm2, as well as spiky oscillations with low or high average p53 levels.

**Conclusions:**

We show that even a simple negative feedback loop is capable of exhibiting the kind of flexible stress-specific response observed in the p53 system. Further, our model provides a framework for predicting the differences in p53 response to different stresses and single nucleotide polymorphisms.

## Background

The tumor suppressor protein, p53, is a transcription factor that regulates the activity of hundreds of genes involved in cell growth and death [1,2]. Over 50% of human cancer cells contain mutations in p53, because of which it has become a key target in cancer research [3]. A wide variety of stress conditions result in the accumulation and activation of p53 - among others: DNA damage, hypoxia, heat shock, nutrient deprivation and oncogene activation. Despite the fact that all these inputs are integrated into a single node, p53, the expression pattern of downstream genes (and hence the physiological response) appears to be specific to each stress. For example, hypoxia invariably leads to apoptosis [4], whereas ribonucleotide depletion leads to reversible cell cycle arrest [5], and UV irradiation can result in either cell cycle arrest or apoptosis depending on the intensity of the damage [6].

How does the regulatory network around p53 retain this exibility even though all inputs converge at a single node? We argue in this paper that the particular design of the p53-Mdm2 feedback loop at the core of this network could be the source of this flexibility. p53 is regulated by other proteins at two levels: its stability (e.g., Pirh2, COP1, Mdm2 decrease its half-life [7-9]), and its activity as a transcription factor (e.g., MdmX, Mdm2 retard its activity [10]). We focus on Mdm2 because (a) Mdm2 null mutants are lethal in early development in mice [11], and (b) Mdm2 directly regulates *both *activity and stability of p53. Mdm2 is an E3 ligase that binds to p53. Mono-ubiquitination of p53 by Mdm2 inhibits its transcriptional activity, while poly-ubiquitination triggers its degradation [12]. In turn, the *mdm2 *gene is activated by p53, thus forming a negative feedback loop [13]. We use a mathematical model of the p53-Mdm2 feedback loop to demonstrate how multiple inputs can be integrated with sufficient discrimination in such a feedback loop to allow diverse, yet specific, output behaviour. Using the model, we can predict which input stresses will produce the stronger p53 response, as well as the effect of single nucelotide polymorphisms (in particular the SNP309 on *mdm2*) on the p53 response.

## Methods

### A model of the p53-Mdm2 negative feedback loop

Our model focuses on the following four concentrations: nuclear-p53, *p*; Mdm2, *m*; Mdm2 mRNA, *m*_*m*_; and the p53-Mdm2 complex, *c*. The temporal dynamics of these components of the model are specified by four differential equations:(1)(2)(3)(4)

The model is provided in SBML format as additional file 1 and in the Biomodels database: http://www.ebi.ac.uk/biomodels-main, model number 1006280000.

**Figure 1:**
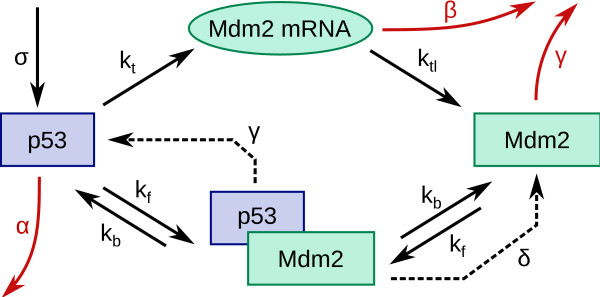
**Schematic representation of the model**. The figure shows, schematically, the components and interactions included in the model. Transcription of *mdm2 *and subsequent translation to the Mdm2 protein are described by the rate constants *k*_*t *_and *k*_*tl*_, respectively. *β *represents the rate of spontaneous degradation of the Mdm2 mRNA. Two rate constants describe complex formation (*k*_*f*_) and breakup (*k*_*b*_). p53 is assumed to be produced at a constant rate *σ*. Degradation of p53 occurs at the rate *δ *when it is Mdm2-mediated, and at the rate *α *when it is Mdm2-independent. Finally, we assume that the Mdm2 degradation rate, *γ*, is independent of whether it is bound to p53 or not.

Figure 1 shows the interactions that correspond to each of the mathematical terms in the above equations. Table 1 lists the parameters of the model and their default values. Some parameters correspond to specific processes (e.g. Mdm2-mediated degradation of p53 (*δ*), activation of *mdm2 *by p53 (*k*_*t*_)). Others are "effective" parameters which model the combined action of several proteins that all affect p53 or Mdm2. For example, *α *models all Mdm2-independent processes which result in the reduction of the active p53 concentration in the nucleus: spontaneous degradation, export out of the nucleus, physical interaction with other proteins, sequestration in the cytoplasm (e.g. by Pirh2), modifications which prevent activity, etc. We also assume that activation of *mdm2 *by p53 has an associated Hill coefficient of 2 (there is a double binding site for p53 at the Mdm2 promotor [14]), and that the half-life of Mdm2 is independent of whether it is free or bound to p53 (ref. [15] shows the latter is true in the absence of stress). Details on how parameter values were chosen are provided in additional file 2.

**Table 1:** Model parameters

Parameter		Default value	Effect of stresses
*σ*	p53 production	1000 nM.hr^-1^	Ribonucleotide depletion ↗
*α*	Mdm2-independent	0.1 hr^-1^	
	degradation/deactivation of p53		
*δ*	Mdm2-dependent	11 hr^-1^	DNA damage, Hypoxia,
	degradation/deactivation of p53		Oncogene, Nitric Oxide: all ↘

*k*_*t*_	Mdm2 transcription	0.03 nM^-1^hr^-1^	Hypoxia ↘
*k*_*tl*_	Mdm2 translation	1.4 hr^-1^	
*B*	Mdm2 mRNA degradation	0.6 hr^-1^	
*γ*	degradation/deactivation of Mdm2	0.2 hr^-1^	DNA damage ↗

*k*_*b*_	p53-Mdm2 dissociation	7200 hr^-1^	
*k*_*D *_= *k*_*b*_/*k*_*f*_	p53-Mdm2 dissociation constant	1.44 nM	Nutlin ↗, DNA damage ↗

**Figure 2:**
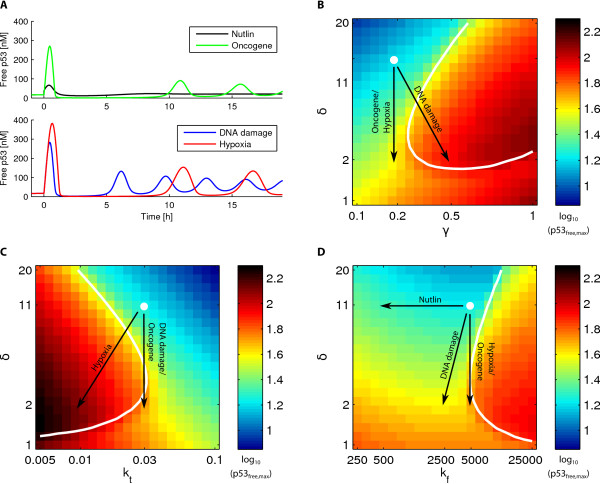
**p53 dynamics**. A: Time dependence of the p53 concentration after application of different cellular stresses at time zero. B-D: The colors show peak concentration of free p53, after the system has settled into a steady-state or stable oscillations (note that the peak level differs from the average only when there are oscillations), as a function of two model parameters. The white line marks where the spikyness (amplitude/average [25]) of p53 oscillations becomes equal to 2 (i.e., where the amplitude becomes twice the average). Thus, spiky oscillations occur to the right of the line in (B) and (D), and to the left in (C). The white dot shows the parameters corresponding to the default resting state (see Table 1). Black arrows illustrate the change in parameters we impose on the system to model the respective stresses.

The numbers show the default value of each parameter, and correspond to the default resting state marked by the white dot in Figure 2. The rightmost column shows which stresses increase (upward arrow) or decrease (downward arrow) the value of a parameter. Details of the choice of parameter values are given in additional file 2.

Most previous models have used an explicit time delay to model transcription and translation (for example, see [16-18]). In these models, the time delays are essential for producing oscillatory behaviour of p53 concentration. Mathematically, the use of explicit time delays converts the equations into delay differential equations which have effectively infinite dimensions and are well known to often exhibit oscillatory behaviour. In contrast our model has no explicit time delay. Thus, the cause of oscillations in our model is completely different; they occur due to the nonlinearities introduced by complex formation between p53 and Mdm2. Other models [19,20] have avoided explicit time delays but used multiple feedback loops, whereas our model uses a single negative feedback loop. [21] has explored a range of different models to reproduce the behaviour under gamma irradiation. Of these, one model, IV, is closest to our model in that it uses a nonlinear degradation of p53 instead of explicit time delays to produce oscillations. However, the molecular mechanism behind this nonlinearity was not discussed. Our model shows that the complex formation between p53 and Mdm2 is sufficient for generating nonlinearities that lead to oscillations. Finally, the main purpose of this paper, to investigate response specificity to different stresses has not, to our knowledge, been studied in any previous model.

## Results and Discussion

### p53 dynamics in the presence and absence of stress

This model system of a negative feedback loop shows plenty of variety in output behaviour. Depending on the values of parameters, the system is capable of steady state solutions with any combination of high or low p53 and Mdm2, as well as oscillations with high or low average p53. Figure 2A shows four examples: a steady state response and oscillations with differing amplitudes and periods. The oscillations are typically spiky, but smooth oscillations can also be generated.

Ideally, we would like to correlate each of these output states of the pathway to specific physiological responses like cell cycle arrest or apoptosis. Clearly, the level of p53 is an important determinant of the response [6,22,23], and the presence or absence of oscillations is also likely to be related to the physiological behaviour [2,24]. When there are oscillations, some downstream genes may respond to the peak p53 level, while others may sense the average level. This depends on the association and dissociation rates of p53 to the relevant operators (this has been discussed in the context of the transcription factor NF-kB in ref. [25], and the same principle would apply to p53). Further, in some cases the response may depend on the activity of p53 as well as its level [22,26]. In sum, not enough information exists to make a precise link between the molecular state and the physiological response. However, it is reasonable to expect that large increases in p53 levels would correlate with a higher incidence of apoptosis, whereas low or moderate increases would correlate with less drastic responses such as cell cycle arrest. Therefore, we have elected to discuss the response in terms of the p53 level. In the figures below we have shown the peak p53 level. Similar figures with average p53 level, and the ratio between free and bound p53 are shown in Figures S3 and S4 of additional file 2.

In the absence of stress, p53 levels are typically maintained quite low. For this, a sufficient Mdm2 level is required to keep the half-life of p53 short. Thus, in a typical "resting" state there is a fairly high turnover of p53. The area shaded in green and blue in Figures 2B-D shows parameter combinations which satisfy these conditions - a low level of p53 and no oscillations. The white dot, the default resting state of the cell, before it is subjected to any stress, was chosen to lie within this blue-green region of parameter space (see Table 1 for the corresponding parameter values). Of course, the precise levels of concentration, and turnover rates, in the resting state can vary from cell to cell, both because of variability in levels of various proteins, as well as the presence of mutations, such as single-nucleotide polymorphisms. We will return to this point later in the paper.

### Specific response to four stresses

The system can be triggered by numerous stresses. We model different stresses as affecting different parameter combinations, as shown in Table 1. Figure 2A shows the diversity in response to different stresses, starting from the same resting state. From Figures 2B-D it already becomes apparent that the level of p53 is more sensitive to changes in *δ *and *k*_*t *_than to the p53-Mdm2 dissociation constant *k*_*D *_= *k*_*b*_*/k*_*f*_. Most stresses, however, affect more than one parameter.

A particularly simple, though "artificial", stress is the introduction of **Nutlin**. Nutlin reduces the binding of Mdm2 to p53, while leaving its other properties unchanged. Nutlin treatment can trigger cell cycle arrest, but not apoptosis [27,28]. This is consistent with our model's prediction that increasing *k*_*D *_(weakening the binding) alone causes a very modest increase in p53 levels (Figures 2A and 2D).

A more common real-world stress is **DNA damage**, which can trigger processes that result in (a) increased auto-ubiquitination of Mdm2, (b) decreased ubiquitination of p53 by Mdm2 and (c) weaker binding of p53-Mdm2 [15,29,30], corresponding in our model to increasing *γ*, decreasing *δ*, and increasing *k*_*D*_. Single-cell experiments have found that irradiation of various types triggers oscillations in p53 levels with a period of 5-6 hours. The parameter changes used to mimic DNA damage stress were chosen such that the response matches the observations of Ref. [31] which found that, in response to ionizing radiation, the first p53 peak occured at around 30 min, the second at 6 hours and the third between 9 and 13 hours. The damping of the amplitude also matches the observations which found the second peak to be around half as high as the first, and the third to be around 2.5 times lower than the first [31]. A similar response is seen when gamma radiation is used to induce DNA damage [21]. When we increase *k*_*D *_and *γ*, while lowering *δ*, corresponding to the molecular processes described above, our model produces an oscillatory solution in accordance with the experimental observations (see Figure 2A).

**Hypoxia **is another stress that increases p53 levels. It is known that under hypoxic conditions, even though p53 accumulates, it does not possess its transactivation property [4,32], i.e., *k*_*t *_is decreased. This means that Mdm2 is down-regulated. Furthermore, hypoxia induces HIF which binds to p53 and prevents degradation [33], which we mimic by decreasing *δ*. Hypoxia does not lead to cell-cycle arrest, suggesting that it typically results in much higher levels of p53. Consistent with this picture, our model yields a stronger response (i.e., oscillations with a bigger amplitude and larger average p53 level) when we apply a hypoxic stress when compared to other stresses with similar fold-changes in parameter values (see Figure 2A).

Deregulated **oncogenes **are another signal that can trigger the p53 pathway. They lead to increased transcription of ARF, which binds to Mdm2 and inhibits its E3 ligase activity [34]. This corresponds to decreasing *δ*, the Mdm2-dependent degradation of p53. The response to this, in our model, is oscillations in p53 but weaker than the response to DNA damage or hypoxia (see Figure 2).

### Predicting the relative strength of the response to different stresses

**Figure 3:**
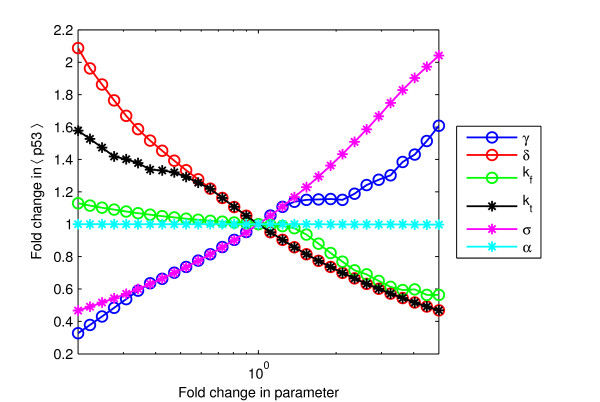
**Sensitivity analysis**. Fold change in average p53 level for fold changes, ranging from 1/5 to 5, in various parameters. For each curve, the corresponding parameter is varied from its default value while keeping all other parameter values fixed. The slope of each curve is a measure of the sensitivity of the p53 level to changes in that parameter.

Figure 3 shows the relative effect on the average free p53 level when each parameter of the model is varied from its default value, keeping the values of all other parameters fixed. The slope of each curve is a measure of how sensitive the p53 level is to changes in the corresponding parameter. There was no necessity to examine variations with respect to *β *and *k*_*tl *_because one can always choose units of time and mRNA concentration such that *β *= 1 and *k*_*tl *_= 1, i.e., changes in *β *or *k*_*tl *_can be mimicked by changes in other parameters.

The average free p53 level is in general much more sensitive to changes in *γ*, *δ*, *k*_*t *_and *ι *than to changes in *α *and *k*_*D*_. The sensitivity with respect to variation of *γ *appears to be very little for values in the range of 1-to 2-fold the default value. This coincides with the onset of oscillations. In contrast, the peak p53 level retains its sensitivity (Figure S4, additional file 2). That is, while the amplitude of oscillations increases significantly the average does not, a feature that arises due to the spikyness of the oscillations. The physiological significance of this is unclear.

Overall, it is clear that stresses that affect only *α *or *k*_*D*_, such as Nutlin, will have the least impact on average p53 level. For other stresses, the relative impact depends on how many of the sensitive parameters they affect. Thus, DNA damage and hypoxia, which each affect two sensitive parameters, result in a relatively stronger response than oncogene deregulation, which only affects one parameter.

### The effect of single nucleotide polymorphisms

**Figure 4:**
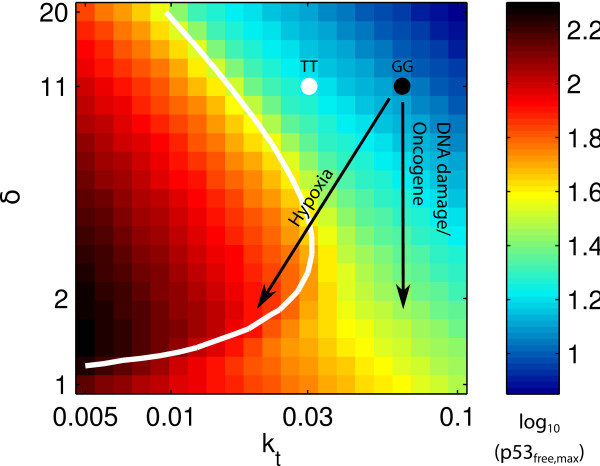
**Effect of the *mdm2 *SNP309**. The color map and the white dot are the same as in Figure 2B-D. In the presence of the *mdm2 *SNP309 G allele, the resting state of the cell is shifted towards higher *k*_*t *_values, as indicated by the black dot. It is not known how much the SNP changes the *k*_*t *_value, but the qualitative behaviour can be predicted: the same stress will lead to a weaker response for GG than for TT.

Our model can also be used to examine the behaviour of certain mutant cell lines. For instance, the G allele of the *mdm2 *single nucleotide polymorphism 309 (SNP309) results in an increased expression of *mdm2 *compared to the T allele [35]. This corresponds to changing the initial resting state from the one in Figure 2C to one with increased *k*_*t *_(i.e., increased rate of transcription of *mdm2*), as illustrated in Figure 4. The figure shows that, in our model, for a given intensity of stress, the response of a cell with the G allele is weaker. This suggests that populations with a higher frequency of the G allele, such as Caucasians (45% TT, 44% TG, 11% GG; as compared to African Americans: 74% TT, 23% TG, 3% GG) should exhibit a lower p53 level in response to stress. Assuming that p53 level correlates with apoptosis, this would suggest a lower incidence of apoptosis, and a higher frequency of tumour formation. This is indeed observed when comparing apoptosis frequency in lymphocyte cell lines from Caucasian and African American donors [36].

### Variability in the p53 response

**Figure 5:**
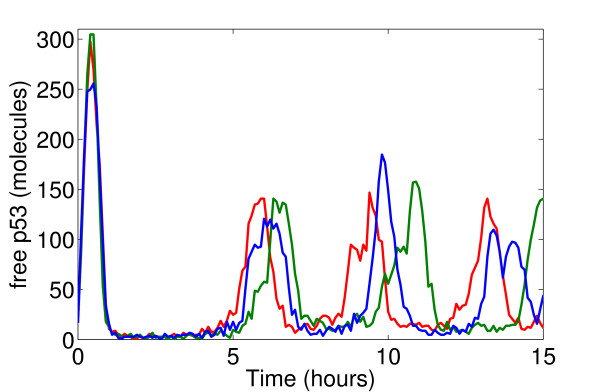
**Effects of noise**. Three independent stochastic runs using the Gillespie algorithm of the model's response to DNA damage. Model parameters are changed at time zero as in Fig. 2. Later peaks in p53 level show more variability than earlier peaks. Molecule numbers were chosen small enough to achieve a visible amount of noise. Using realistic numbers makes the stochastic simulations indistinguishable from deterministic behaviour (see text and Figure S6 in additional file 2).

Single-cell measurements of p53 oscillations in response to various types of irradiation exhibit a fair amount of variability in the response across different cells [21,31]. The deterministic simulations we have done cannot address this issue, so stochastic simulations are required. However, the numbers of p53 and Mdm2 molecules are typically very large: Measured levels of p53 range from 17,000-200,000 molecules in different cell lines [17,37], and the resting level of p53 in our model, ≈ 100 *nM*, corresponds to 50,000 molecules per cell (assuming the cell is a sphere of radius around 6 *μm*). Such high numbers mean that the noise due to stochasticity in production and degradation of molecules is very small. Thus, the result of stochastic simulations of our model using the standard Gillespie algorithm [38] are indistinguishable from the deterministic simulations. It is possible that there are other sources that result in a higher noise in cells. If we increase the noise in our Gillespie simulations in an ad-hoc manner (we do this by arbitrarily assuming a 500-fold smaller cell volume, thereby decreasing the overall numbers of both p53 and Mdm2 proportionally; other stochastic models [39] also seem to work with similarly low numbers of p53 despite the measurements) then we observe that the first peak position is quite robust to noise, the second peak position varies a little more, while the third peak position varies significantly (see Fig. 5). This is exactly what has been observed in single-cell experiments of the p53 response to ionizing radiation [31]. On the basis of these stochastic simulations we hypothesize that:

1. The variability in p53 response observed in [21,31] must originate from sources other than stochasticity in the production and degradation of molecules, because the numbers of involved molecules are rather large.

2. Increasing amounts of noise are likely to introduce more variability in the position of later p53 peaks than in earlier peaks, as observed.

However, a proper analysis of these hypotheses requires a better knowledge of which sources of noise underly the variability observed, so that they can be modelled accurately.

## Conclusions

### Predictions from the model

The sensitivity analysis in Figure 3 shows which parameters most affect the p53 level in our model. Combining this information with a knowledge of which parameters are affected by different stresses provides predictions about which stresses will affect the p53 level the most. One specific prediction is that around the onset of oscillations, changes in *γ *result in large changes in peak p53 levels but hardly any change in average p53 levels.

The analysis also leads to a prediction of reduced p53-dependent apoptosis in populations which have an increased frequency of the G allele of the *mdm2 *SNP309 - a prediction that is confirmed by observations. In addition, if the increase in *k*_*t *_due to the SNP is sufficient, then although p53 will be upregulated in response to stress, oscillations will not occur (as can be seen from Figure 4). This effect has also been observed experimentally [35]. The same analysis method can be used to predict the effect of other SNPs as soon as one knows which parameters (i.e., which molecular processes) they affect.

Finally, we note that the temporal dynamics of the p53 response to different stresses are also predictions of the model that can be tested experimentally. To our knowledge, single cell experiments examining the p53 dynamics in response to hypoxia or oncogene deregulation have not been done. Our model predicts that oscillations should be observed in both cases, which tend to have longer time periods than in response to DNA damage and with a particularly distinct time delay (and reduction of amplitude) between the first and second peaks.

### Extending the model

Our model could eventually be extended to cover other stresses that trigger a p53 response as more data becomes available. Nitric oxide (NO) is a free radical produced in inflamed tissue which can trigger the p53 pathway by phosphorylating p53 and thereby inhibiting its Mdm2-mediated degradation [40]. Another example is ribonucleotide depletion: cells suffering this undergo a reversible p53-dependent cell cycle arrest [5]. How this happens has not been fully worked out, but a hypothesis exists: the depletion could cause a redistribution of p53 from cytoplasm to the nucleus, where it can be transcriptionally active [5]. Finally, heat shock can also trigger p53 but the picture is rather complex and indecisive, involving various chaperones and heat shock proteins [41-43]. Other directions to extend the model are of course to include other feedback loops and essential players in p53 regulation, such as Wip1 [20] and MdmX [10], and to model the connection between p53 levels and physiological behaviour more accurately as has been done for cell cycle arrest in ref. [44].

Overall, we have shown that this kind of negative feedback loop, consisting of a relatively slow transciptional activation on one leg of the loop, and an inhibition based on fast complex formation on the other, can be designed to respond specifically to a number of different input triggers. This kind of negative feedback loop also occurs in another important signalling pathway that is triggered by hundreds of input signals, namely NF-*κ*B signalling in the immune system [45]. NF-*κ*B is a transcription factor that controls hundreds of downstream genes. It activates production of I*κ*B*α*, which binds to and inhibits the action of NF-*κ*B [46]. The resultant negative feedback loop exhibits spiky oscillations [25,47] similar to the kind we observe in the model presented here. Thus, our results might also have relevance beyond p53.

## Authors' contributions

AH, SK and MHJ designed, implemented and analyzed the model, and wrote the paper. All authors read and approved the final manuscript.

## Supplementary Material

Additional file 1 **Model in SBML format**. This file provides the p53-Mdm2 model we use in SBML format. Click here for file

Additional file 2 **Supplementary information**. This file provides additional information and figures to supplement the main text. Click here for file
