# Supplementary material for: Stress-specific response of the p53-Mdm2 feedback loop
Source: BMC Syst Biol. 2010 Jul 12;4:94. doi: 10.1186/1752-0509-4-94 (PMC2913930; doi:10.1186/1752-0509-4-94)
Supplement: Additional file 2 — Supplementary information. This file provides additional information and figures to supplement the main text. [file 1752-0509-4-94-S2.PDF]

Supplementary information for "Stress-specific response of the p53-Mdm2 feedback loop"

Alexander Hunziker[1], Mogens H Jensen[1] and Sandeep Krishna*[1,2]

[1]Center for Models of Life, Niels Bohr Institute, Copenhagen, Denmark
[2]National Centre for Biological Sciences, Bangalore, India

Email: Alexander Hunziker - hunziker@nbi.dk; Mogens H Jensen - mhjensen@nbi.dk; Sandeep Krishna - sandeep@nbi.dk;

*Corresponding author

The model

Our model focuses on the following four concentrations: nuclear-p53, p; Mdm2, m; Mdm2 mRNA, m_m; and the p53-Mdm2 complex, c. The temporal dynamics of these components of the model is specified by four ordinary differential equations:

$$\frac{dp}{dt} = \sigma - \alpha p - k_f pm + k_b c + \gamma c \quad (1)$$

$$\frac{dm_m}{dt} = k_t p^2 - \beta m_m \quad (2)$$

$$\frac{dm}{dt} = k_{tl} m_m - k_f pm + k_b c + \delta c - \gamma m \quad (3)$$

$$\frac{dc}{dt} = k_f pm - k_b c - \delta c - \gamma c \quad (4)$$

Parameter values

There are 9 parameters in these equations. Some can be fixed or constrained using data from literature:
1. p53 does not measurably degrade over 120 min in the absence of Mdm2 [1]. Therefore, its Mdm2-independent degradation rate, α, is very low. We took $\alpha = 0.1hr^{-1}$, corresponding to a half life of 7.5 hours. In any case, as shown in Fig. 3 of the main text, varying α by upto 5-fold up or down makes no difference to the behaviour of the model.
2. The Mdm2 mRNA half-life has been measured to be 1–2 hours [2,3], so we fixed $\beta = 0.6hr^{-1}$ corresponding to a half-life of a little over 1 hr.
3. k_b has been measured in ref. [4] to be 1.4 to 2 s^{-1}. We therefore fix $k_b = 7200hr^{-1}$.
4. The dissociation constant $k_D \equiv k_b/k_f$ of the p53-Mdm2 complex has been reported to be various numbers, ranging from 100 molecules [5] (around 0.2 nM[1]), to 60 nM [4] to 700 nM [4]. This implies k_f should lie in the range 10–36,000 $nM^{-1}hr^{-1}$.
5. The Mdm2-dependent half-life of p53 was measured to be less than 15 min [1]. We therefore explored δ in the range 1–20 hr^{-1}, corresponding to a half-life in the range 2–40 min.

[1] $1nM \approx 0.6V$ molecules/cell, where V is the volume of the cell in μm^3. As a default, we assume a cell volume of around 830 μm^3, corresponding to a sphere of radius around 6 μm. This gives 1nM=500 molecules/cell.

Knowing the levels of p53 and Mdm2 in the absence of stress can be used to constrain the values of σ, γ and the product $k_t k_{tl}$ as follows:
The level of p53 in cells has been measured to be in the range 17,000–200,000 molecules in various cell lines [6, 7], which is around 35–400 nM (assuming as before 1nM=500 molecules/cell). We aimed for a total p53 level of 100 nM in the absence of stress. Further, we aimed to have enough Mdm2 such that 80-90% of the total p53 is in complex with Mdm2. This means the free p53 should be around 20nM. And free Mdm2 level should be around 9 or 10 times k_D (so that $c/p^{tot} = m/(m + k_D) \approx 0.9$). As a starting point, using we assumed this level would be around 10–20 nM, so the total Mdm2 would also be around 100 nM, i.e., the complex dominates both p53 as well as Mdm2 levels. Now we can obtain two relations connecting as yet unkown parameters to known ones:
(a) In steady-state, production and degradation of p53 should balance, so

$$\sigma \approx \delta c$$

Taking a value of $\delta = 10$ or $11\ hr^{-1}$, in the middle of the range we wanted to explore, and with $c = 80$ to 90 nM (because 80–90% of p53 is bound), we get an estimate of $\sigma \approx 1000\ nM.hr^{-1}$, which is what we use as the default value.
(b) Production and degradation of Mdm2 should also balance:

$$\frac{k_t k_{tl}}{\beta} p^2 = \gamma m^{tot}$$

Taking $m^{tot} \approx 100nM$ and $p \approx 20nM$, this leads to

$$k_t k_{tl} = 0.2\gamma. \tag{5}$$

With α, β, k_b and σ fixed as explained above, we varied other parameters to find oscillations similar to those described in response to DNA damage in [8, 9]. k_f and δ were varied in the ranges mentioned above, and a very wide range of γ was explored (0.01–100 hr^{-1}), with $k_t k_{tl}$ set according to Eq. 5. Fig. 2 in the main text and Figs. S1–S3 show the regions where interesting behaviour was observed. We found that $k_f = 2500nM^{-1}hr^{-1}, \delta = 2hr^{-1}, \gamma = 0.5hr^{-1}$ produced oscillations of the correct time period and characteristics.

From this state we looked in the direction of increasing k_f and δ, and decreasing γ, for the default "resting state" of the cell (because DNA damage was to be modelled by decreasing k_f and δ, and increasing γ). We wanted the resting state to have:
1. no oscillations
2. a low level of p53, around 100 nM
3. a high enough level of Mdm2 such that 80–90% of p53 is bound to Mdm2

We obtained such a state with $k_f = 5000nM^{-1}hr^{-1}, \delta = 11hr^{-1}, \gamma = 0.2hr^{-1}$, and $k_t k_{tl} = 0.04nM^{-1}hr^{-2}$. (Note that the values of k_t and k_{tl} do not have independent significance. Increasing one can be compensated by decreasing the other, which will simply change the mRNA level. Since we are focussing on protein levels and not mRNA level, we arbitrarily set $k_t = 0.03nM^{-1}hr^{-1}$ and $k_{tl} = 1.4hr^{-1}$.) With these parameters, in steady-state, we obtain $p \approx 17nM, m \approx 8nM$ and $c \approx 91nM$. Thus, $p^{tot} \approx 108nM$ and $c/p^{tot} \approx 84\%$, which is what we were aiming for.

Parameter changes to mimic stresses

Nutlin prevents the binding of p53 to Mdm2, so we model this by decreasing k_f 10-fold. Given that k_b is kept constant this implies a 10-fold increase in the dissociation constant, k_D.

As explained in the main text, we model DNA damage stress by decreasing δ, increasing γ and increasing k_D from the resting state. We found, as described earlier, that choosing $\delta = 2hr^{-1}$, $\gamma = 0.5hr^{-1}$ and

$k_f = 2500 nM^{-1} hr^{-1}$ produced oscillations that matched those described in [8]. They observe the first peak at around 30 min, the second at 6 hrs and the third in the range 9-13 h. The amplitude of the second peak is around 50% of the first, while the amplitude of the third is around 40% of the first.

Oncogene regulation was modelled as a decrease in δ only. Therefore, we chose the same change as for DNA damage: $\delta = 2hr^{-1}$. The same change was used in modelling hypoxia along with, as explained in the main text, a reduction of k_t, 3-fold, to 0.01 $nM^{-1}hr^{-1}$. The precise amounts by which to change parameters for each stress of course depend on the intensity of the stress, so we can at best pick out the different directions in parameter space along which the cell is moved, upon being subject to different stresses.

Finally, in order to model the presence of the G allele at SNP309, we used a resting state with a higher k_t value. Stresses applied to these cells were modelled using the same parameter changes as above. Again, the precise amount k_t is increased by the G allele is unknown, so we chose, in Fig. 4, to double the default value in order to make obvious the differences in response to stresses.

Implementation

The differential equations were integrated using (i) MATLABs ode45 and ode15s solvers, and (ii) an adaptive step-size fourth order Runge-Kutta algorithm implemented in C++. The model is also available in SBML format as part of this supplementary material.

In calculating long-timescale features of p53 dynamics (i.e., after initial transients) as a function of various parameters, each run was made for 200 hours. The initial 100 hours of each run were discarded, and the rest used to determine maximum, minimum and average free p53 levels. From this the spikyness and fold-changes (for the sensitivity analysis of Fig. 3 in the main text) were calculated. The average separation between positions of maximum free p53 was taken as an estimate of the time period where oscillations were detected.

Time period and spikyness of oscillations

Fig. S1 shows the time period and spikyness of oscillations as a function of two parameters. Spikyness is defined as (max-min)/average [10]. The white lines correspond to the contour of spikyness=1. Fig. S2 shows three examples of time series of free p53 and free Mdm2 levels to illustrate the kinds of oscillatory and steady-state behaviour generated by the model over long time scales.

p53 levels

In Fig. S3 we show the peak free p53 level, the average free p53 level, and the ratio of free to bound p53 as a function of two parameters. The white lines correspond to the spikyness=1 contour obtained from the previous calculations shown in Fig. S1. Fig. S4 shows the sensitivity analysis, as in Fig. 3 of the main text, for peak free p53 level, the average free p53 level, and the ratio of free to bound p53. Fig. S5 shows the dynamics of the free p53 level, total p53 level (free+bound) and ratio of free to bound p53, for the four different stresses that were shown in Fig. 2 of the main text.

Stochastic simulations

The stochastic simulations were done using the Gillespie algorithm [11], implemented in C++, applied to the model equations. In the standard simulations, 1nM was equated to 500 molecules per cell (which corresponds to assuming a cell volume of around 830 μm^3, equal to that of a sphere of radius around 6 μM) in converting the second-order reaction rates to probabilities per unit time required by the Gillespie algorithm. These gave results essentially indistinguishable from the deterministic runs (see Fig. S6). In order to increase the amount of noise in the runs, we arbitrarily reduced the assumed cell volume such that 1nM corresponded to 1 molecule (for this to be correct the cells would have to be as small as typical bacterial cells). See the red curve in Fig. S6. This decreases the number of molecules thereby increasing the noise in the system without

altering the molecular processes and their relative rates. The runs shown in Fig. 5 in the main text were made using this noise level.

References

1. Barboza JA, Iwakuma T, Terzian T, El-Naggar AK, Lozano G: **Mdm2 and Mdm4 loss regulates distinct p53 activities**. *Mol. Cancer. Res.* 2008, **6**:947–954.

2. Hsing A, Faller DV, Vaziri C: **DNA-damaging Aryl Hydrocarbons Induce Mdm2 Expression via p53-independent Post-transcriptional Mechanisms**. *J. Biol. Chem.* 2000, **275**:26024–26031.

3. Mendrysa SM, McElwee MK, Perry ME: **Characterization of the 5' and 3' untranslated regions in murine mdm2 mRNAs**. *Gene* 2001, **264**:139–146.

4. Schon O, Friedler A, Bycroft M, Freund SMV, Fersht AR: **Molecular Mechanism of the Interaction between MDM2 and p53**. *J. Mol. Biol.* 2002, **323**:491–501.

5. Proctor CJ, Gray DA: **Explaining oscillations and variability in the p53-Mdm2 system**. *BMC Sys. Biol.* 2008, **2**:75.

6. Ma L, Wagner J, Rice JJ, Hu W, Levine AJ, Stolovitzky G: **A plausible model for the digital response of p53 to DNA damage**. *Proc. Natl. Acad. Sci. (USA)* 2005, **102**:14266–14271.

7. Wang YV, Wade M, Wong E, Li YC, Rodewald LW, Wahl GM: **Quantitative analyses reveal the importance of regulated Hdmx degradation for p53 activation**. *Proc. Natl. Acad. Sci. (USA)* 2007, **104**:12365–12370.

8. Hamstra DA, Bhojani MS, Griffin LB, Laxman B, Ross BD, Rehemtulla A: **Real-time Evaluation of p53 Oscillatory Behavior In vivo Using Bioluminescent Imaging**. *Cancer Res.* 2006, **66**:7482–7489.

9. Geva-Zatorsky N, Rosenfeld N, Itzkovitz S, Milo R, Sigal A, Dekel E, Yarnithky T, Polak P, Liron Y, Kam Z, Lahav G, Alon U: **Oscillations and variability in the p53 system**. *Molecular Systems Biology* 2006, **2**.

10. Krishna S, Jensen MH, Sneppen K: **Minimal model of spiky oscillations in NF-kB signalling**. *Proc. Natl. Acad. Sci. (USA)* 2006, **103**:10840–10845.

11. Gillespie DT: **Exact Stochastic Simulation of Coupled Chemical Reactions**. *J. Phys. Chem.* 1977, **81**:2340–2361.

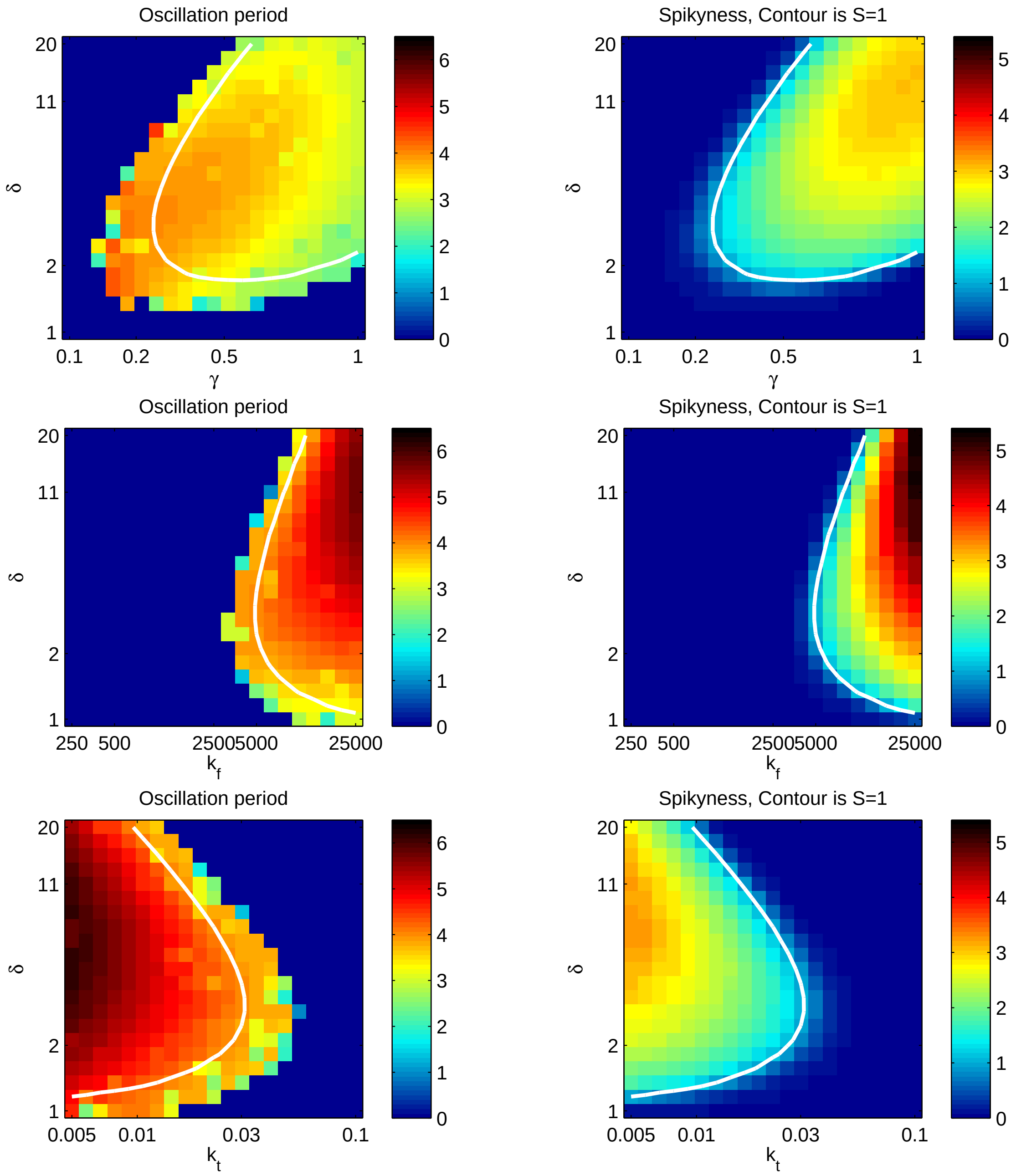

Figure S1: Left panels: time period of oscillations (in hours). No sustained oscillations occur in dark blue areas. Right panels: spikyness, (max-min)/average, of free p53 levels. White curves in all plots correspond to the contour of spikyness=1.

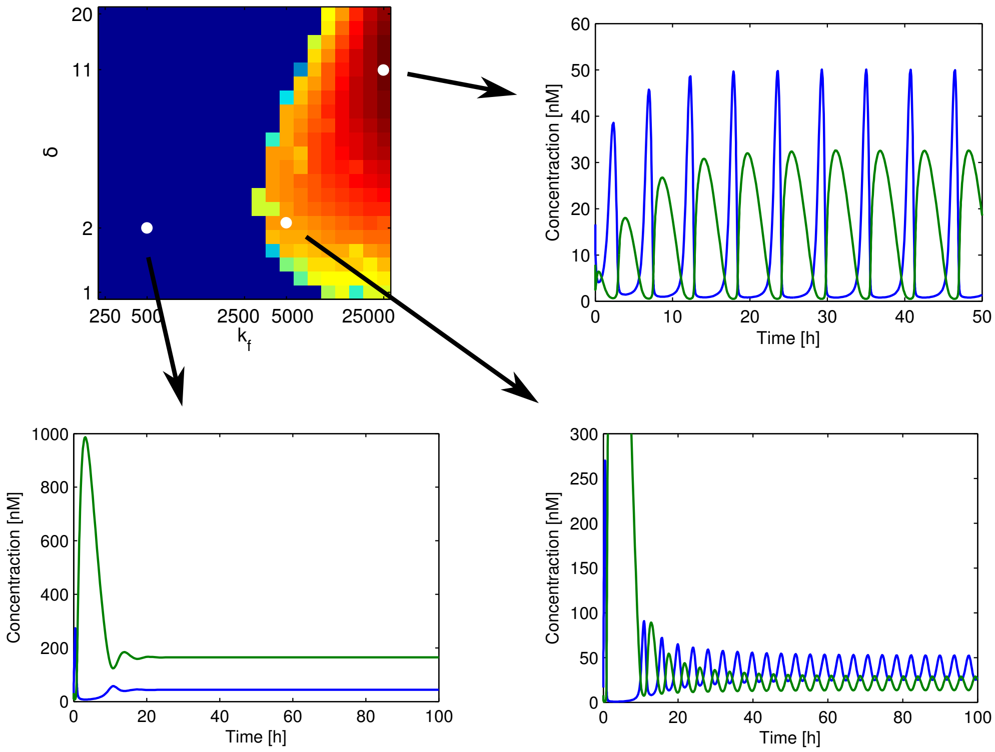

Figure S2: Three time series showing spiky, smooth and damped oscillations in free p53 (blue). Also shown is free Mdm2 (green). Initial conditions were taken to be the steady-state values in the default resting state, as defined in the main text.

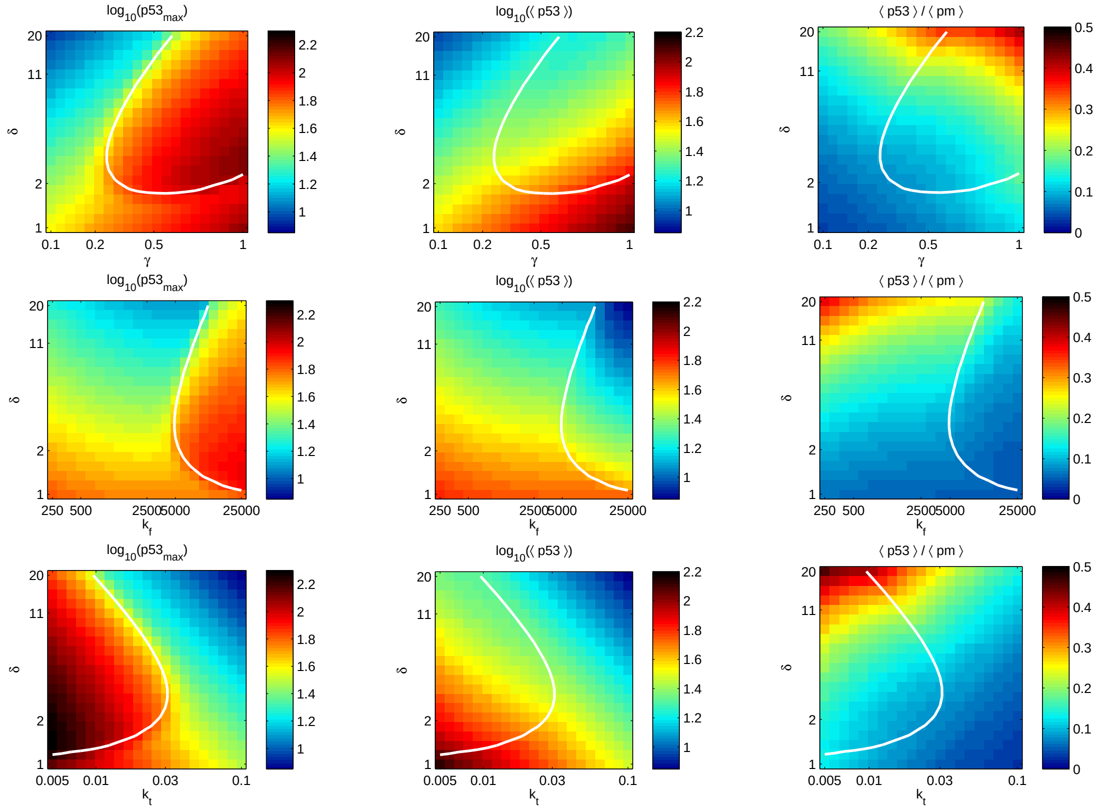

Figure S3: Peak levels of free p53 (left panels), average free p53 (middle) and the ratio of free to bound p53 (right panels), as a function of various parameters. White lines correspond to the contour of spikyness=1 for free p53. The left panels are the same as the ones shown in Fig. 2 of the main text.

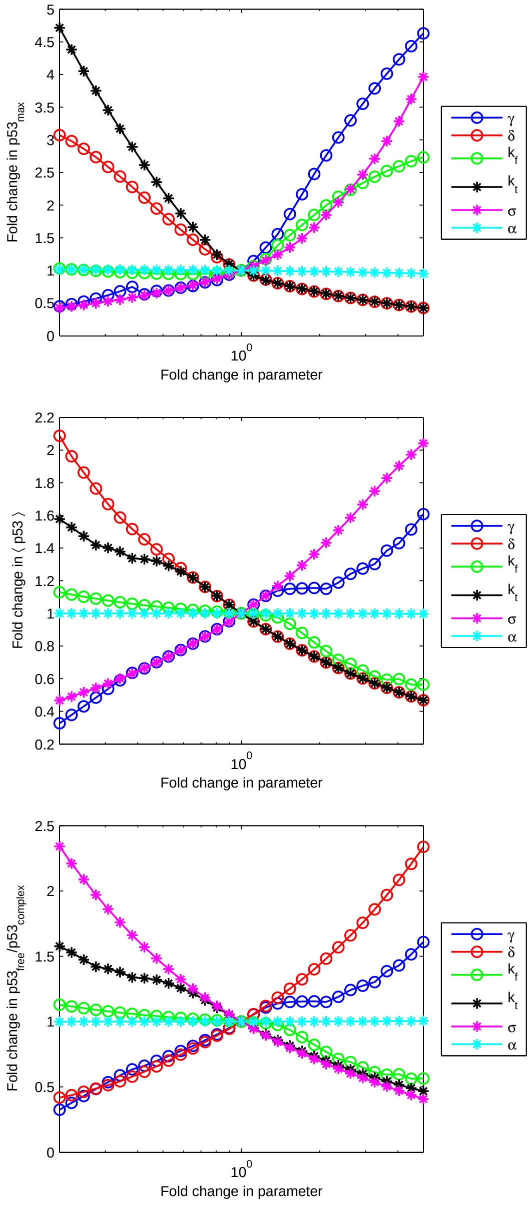

Figure S4: Change in peak free p53 level (top), average p53 level (middle) and ratio of free to bound p53 (bottom), as a function of changes in parameters. For each curve, the specified parameter is varied keeping all others fixed at their default values as specified in Table 1 of the main text. The middle panel is identical to Fig. 3 of the main text.

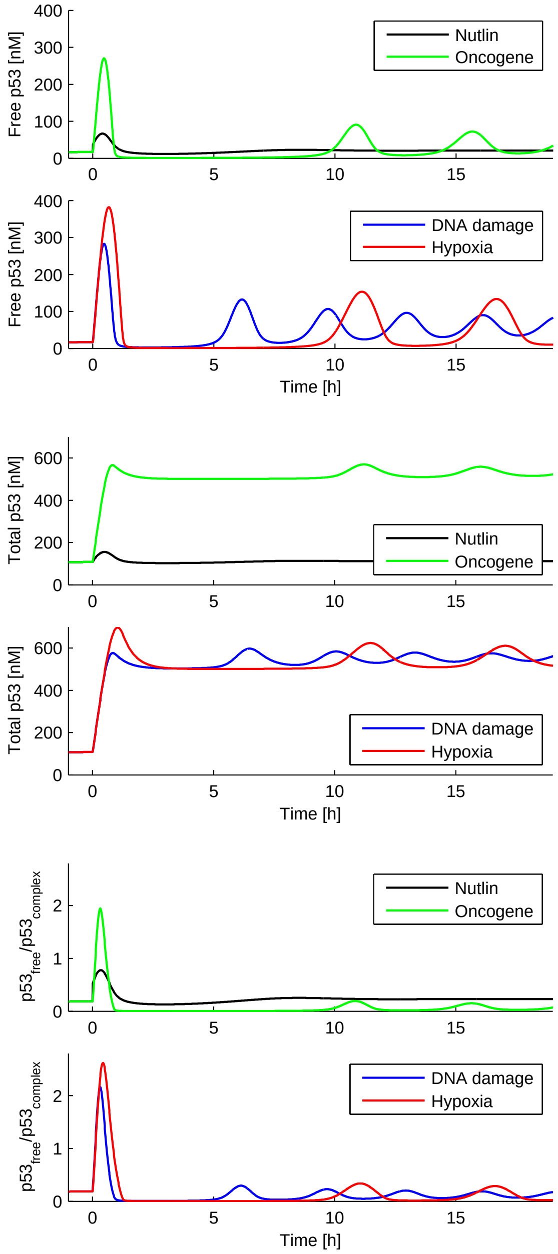

Figure S5: Temporal dynamics of free p53 (top two), total p53 (middle two), and ratio of free to bound p53 (bottom two), in response to four stresses. The top two panels are identical to Fig. 2A of the main text.

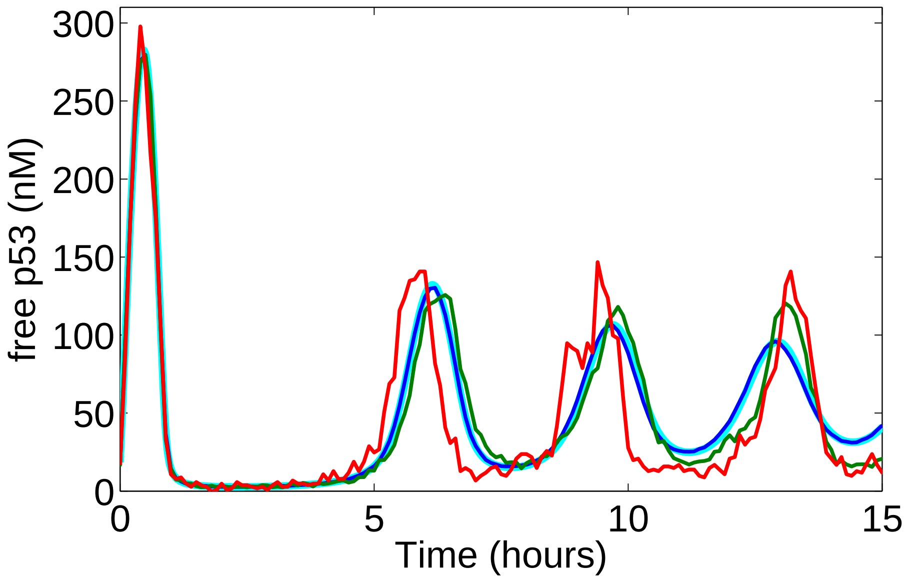

Figure S6: Stochastic simulations of the p53-Mdm2 model. The light blue curve shows the behaviour of free p53 in the deterministic model in response to DNA damage (the curve is identical to the one in Fig. 2A of the main text). A Gillespie simulation with 1nM=500 molecules/cell (dark blue curve) is indistinguishable from the deterministic result. Assuming a smaller volume of the cell increases noise in the Gillespie simulations (green curve: 1nM=10 molecules/cell; red curve: 1nM=1 molecule/cell), especially in the position of the later peaks. The red curve is the same as that shown in Fig. 5 in the main text.
